# Sigmoid Volvulus in the Setting of Type 4 Hiatal Hernia: An Uncommon Presentation and Literature Review

**DOI:** 10.7759/cureus.63595

**Published:** 2024-07-01

**Authors:** Lovingly M Ferrer Ocampo, Jennifer Lin, Leah Donnatien, Gurpreet Singh, Robert Lincer

**Affiliations:** 1 Biomedical Sciences, Touro College of Osteopathic Medicine, Middletown, USA; 2 General Surgery, Garnet Health Medical Center, Middletown, USA

**Keywords:** giant hiatal hernia, volvulus, sigmoid volvulus, hiatal hernia, type iv hiatal hernias

## Abstract

Hiatal hernias occur when intra-abdominal contents protrude into the diaphragmatic opening. Of the four classifications, Type 4 hiatal hernias are the most rare and severe. They develop from herniation of the gastroesophageal junction and abdominal viscera other than the stomach into the thoracic cavity. The resulting increase in intrathoracic pressure can cause a wide variety of symptoms on presentation and potentially lead to misdiagnosis. We present a rare case in which a 78-year-old woman presented with nonspecific symptoms and was diagnosed with incarcerated Type 4 hiatal hernia with sigmoid volvulus. We also report a literature review from 2015 to emphasize the importance of recognizing diverse symptomatic presentations in complex Type 4 hiatal hernias and the need for a comprehensive evaluation, as early detection and prompt intervention are essential in preventing life-threatening complications.

## Introduction

Hiatal hernias can be described as a shifting of abdominal contents into the diaphragmatic opening. They can be classified into four categories of increasing severity and rarity. Type 1 is often referenced as a “sliding” hiatal hernia; these are the most common and comprise 95% of hiatal hernia cases [1]. They are called “sliding” hiatal hernias because the gastroesophageal (GE) junction slides up into the thoracic cavity. A typical symptom is acid reflux, which is usually treated effectively with lifestyle modifications and antacids or proton pump inhibitors [2]. Types 2 and 3 are paraesophageal hernias, meaning that the stomach partially protrudes through the hiatus instead of being fully contained underneath the diaphragm and now resides next to the esophagus. The difference between Types 2 and 3 is the location of the GE junction: in Type 2, the GE junction is in its normal anatomical position, but in Type 3, the GE junction has herniated into the thoracic cavity. Paraesophageal hernias are less common and account for less than 5% of the total number of hiatal hernias [3]. Typical symptoms can also include reflux, which can sometimes present with difficulty swallowing due to GE dysfunction. If conservative measures fail, surgical procedures such as fundoplication, gastropexy, or closure of the hiatal defect can be considered [3]. The most severe and uncommon hiatal hernia is Type 4, which can be defined as herniation of both the GE junction and abdominal viscera, not including the stomach, into the thoracic cavity. Because of the increased intrathoracic pressure, patients present with a multitude of symptoms, including chest pain, shortness of breath, and constipation, which often lead to misdiagnosis. Early intervention and detection may be crucial for these patients because of the possibility of life-threatening complications, such as those seen in the case we present [4]. By reporting a rare case of an incarcerated Type 4 hiatal hernia with sigmoid volvulus and with a comprehensive literature review reporting from 2015 to the present, we hope to emphasize the diversity of symptomatic presentations in Type 4 hiatal hernias and the need for comprehensive evaluation.

## Case presentation

A 78-year-old woman presented to the emergency department (ED) with sudden and severe abdominal pain, lightheadedness, and constipation that had lasted for three days. On route to the ED, she was diaphoretic, hypotensive, and bradycardic. Her vitals were taken on arrival: blood pressure, 104/55; pulse, 93; temperature, 94.5 (rectal); respiration rate, 20; and SpO_2_, 93%. She reported abdominal pain, nausea, vomiting, and dizziness but denied having fever, chills, or diarrhea. She reported no urinary frequency, urgency, or dysuria. She denied having numbness, tingling, or a headache. The physical examination was significant for diffuse abdomen tenderness with positive rebound tenderness and guarding of the epigastric region. The abdomen was soft and only moderately distended. Laboratory results on admission showed an elevated white blood cell count of 21.4 with a neutrophil predominance, as demonstrated by the absolute neutrophil count of 18,511. Her basic metabolic panel (BMP) was significant for low CO_2_, her glucose was elevated at 158, and she showed signs of an acute kidney injury. Her blood urea nitrogen (BUN) was 48, and creatinine was 1.92, with a low estimated glomerular filtration rate of 26.4. She appeared to be acidotic with an increased lactic acid level of 2.5. Her troponin and electrocardiogram were within normal limits.

**Figure 1:**
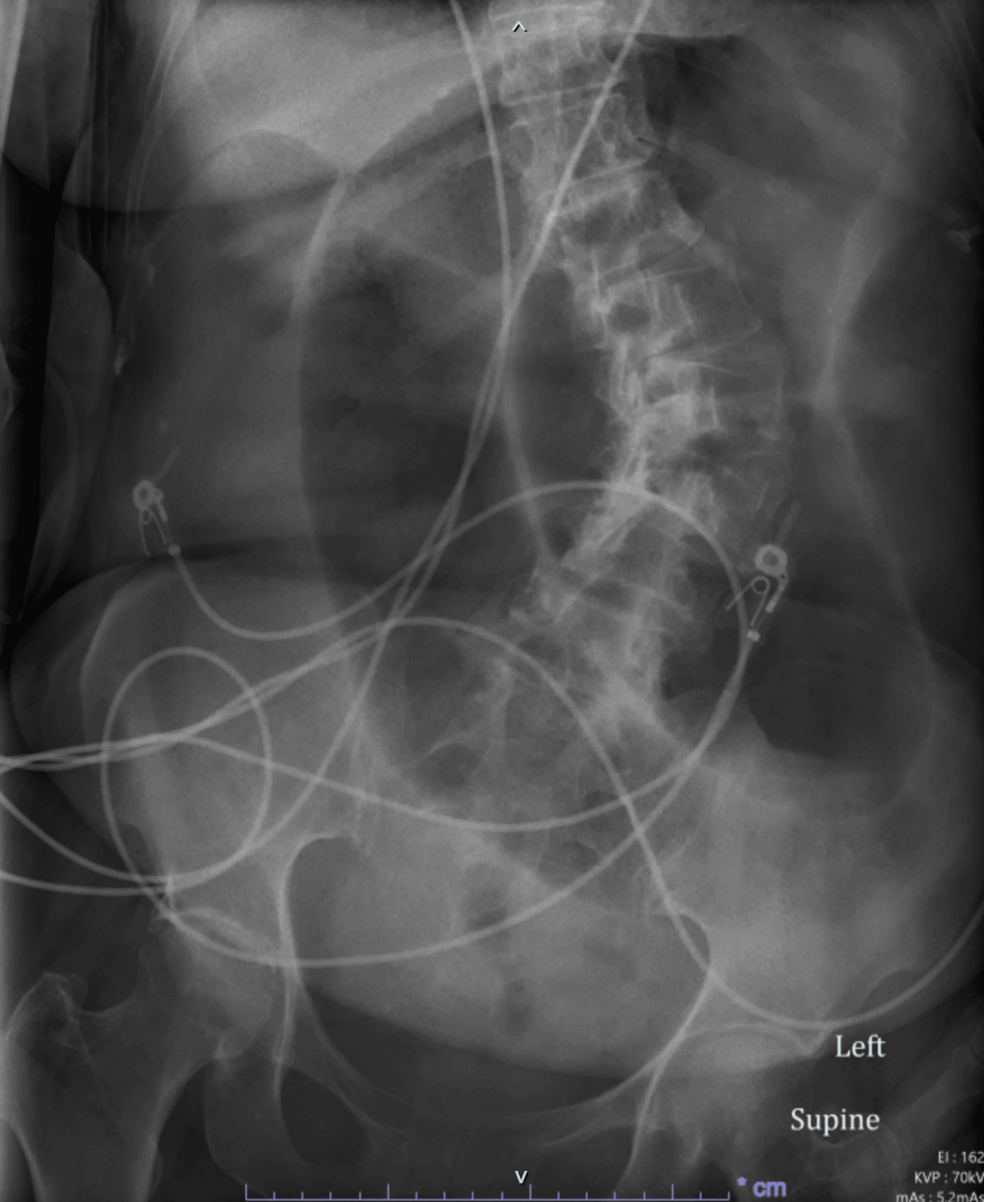
X-ray of the abdomen showing dilated bowel loops, possibly representing a redundant sigmoid colon or sigmoid volvulus.

Plain abdominal radiography was significant for a large hiatal hernia and dilated bowel loops, possibly representing a redundant sigmoid colon or sigmoid volvulus (Figure 1)**.**

**Figure 2:**
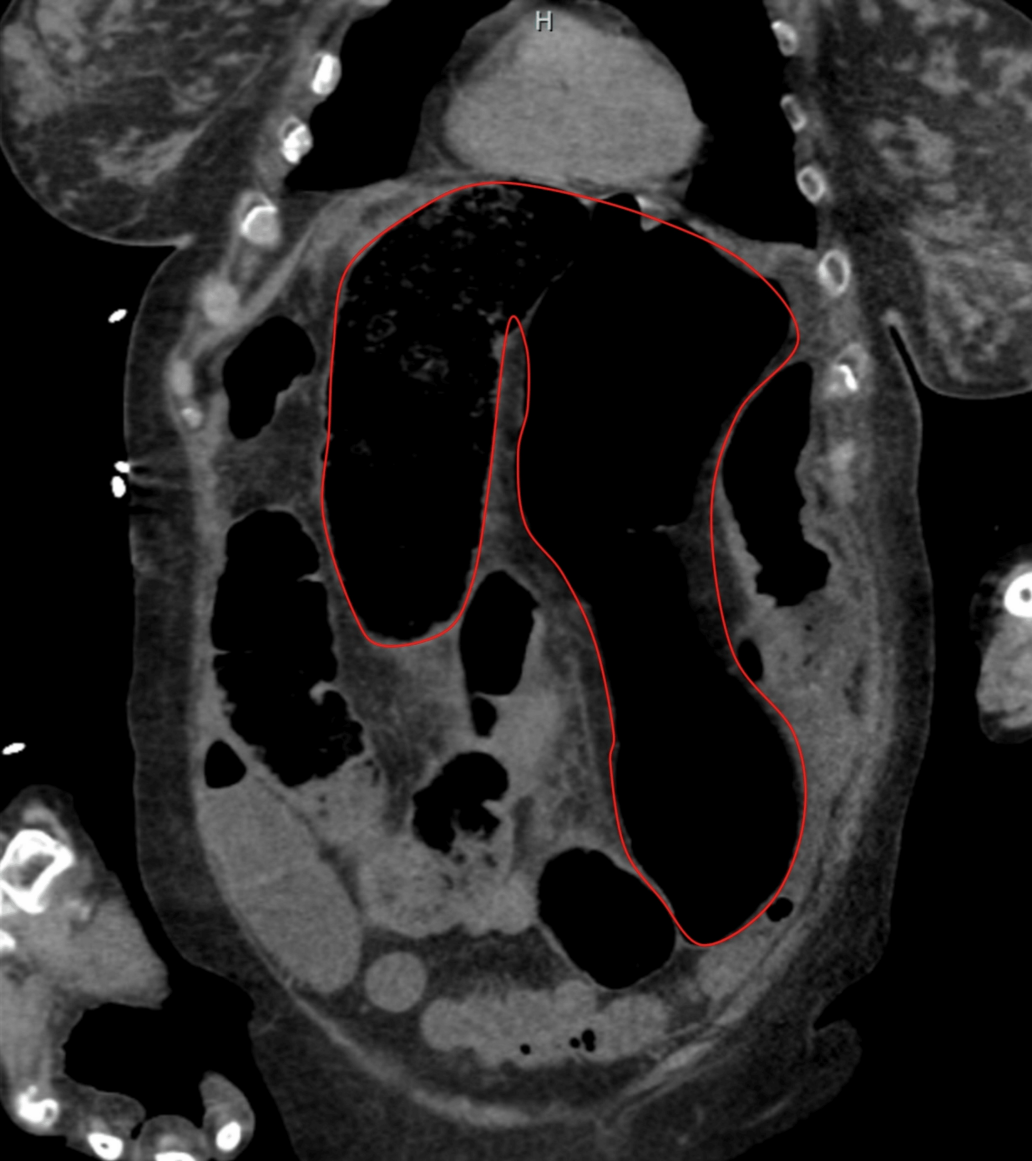
AP view of CT abdomen and pelvis without contrast showing a dilated bowel consistent with sigmoid volvulus, exhibiting the classic "coffee bean" sign, which is delineated in red. AP: anterior-posterior; CT: computed tomography

**Figure 3:**
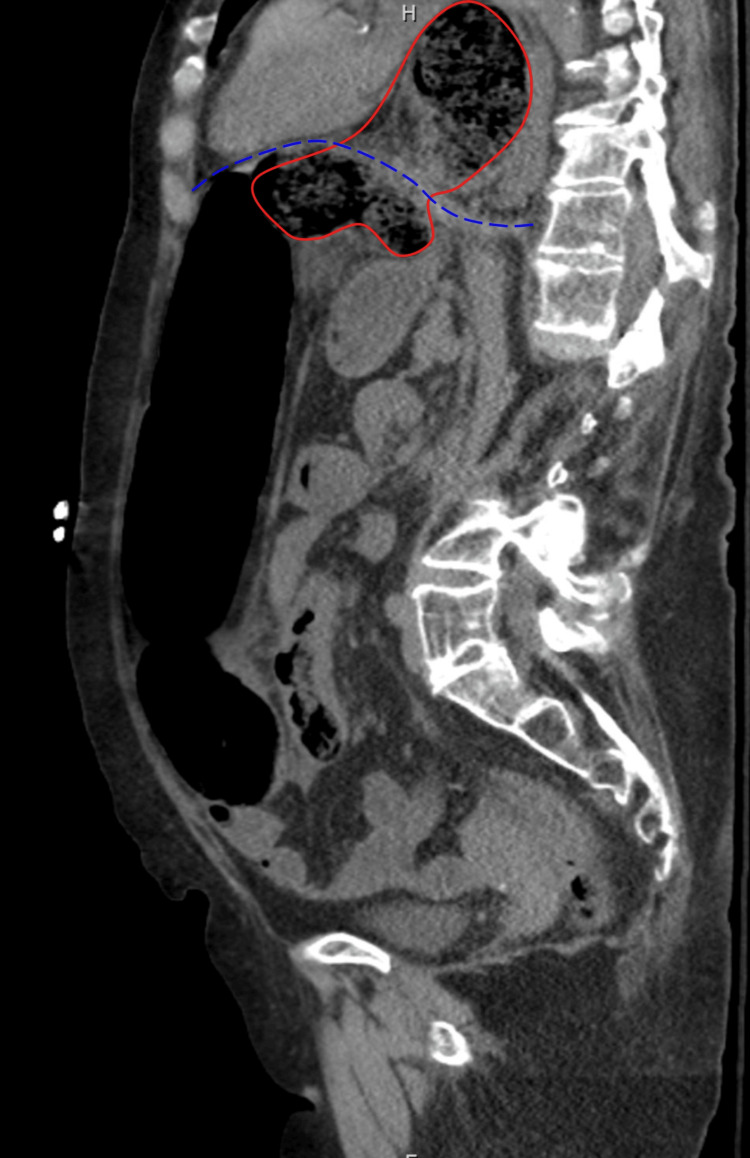
A sagittal view of the CT abdomen and pelvis shows a dilated bowel anteriorly (left) and a necrotizing bowel herniating into the thoracic cavity. The incarcerated apex of the sigmoid volvulus is shown, delineated in red. The diaphragm level is delineated by the dashed blue line. CT: computed tomography

**Figure 4:**
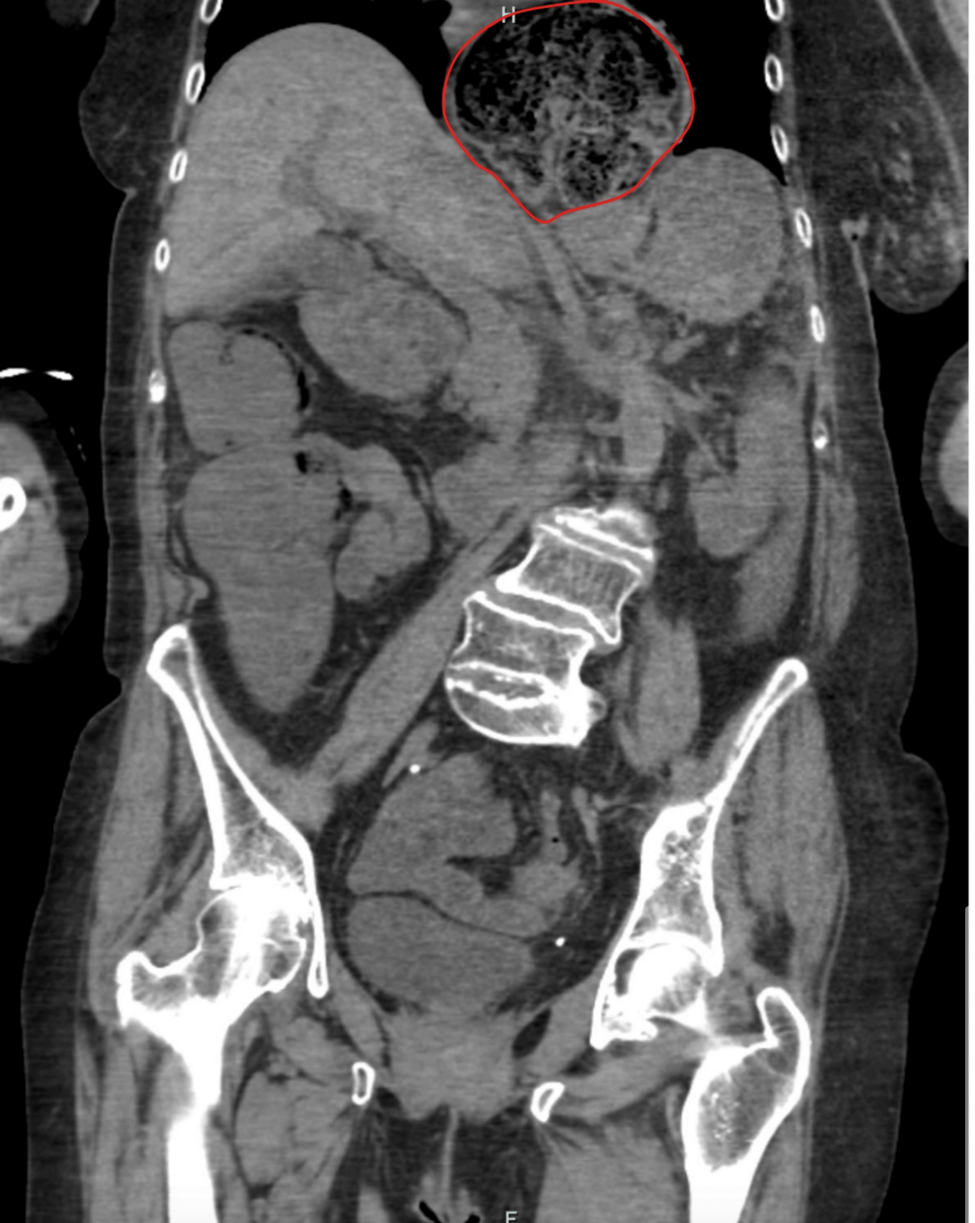
AP view of the CT abdomen and pelvis without contrast showing compression at the level of the aortic hiatus due to herniation of the apex of the sigmoid volvulus. The herniated bowel above the level of the diaphragm is delineated in red. AP: anterior-posterior; CT: computed tomography

**Figure 5:**
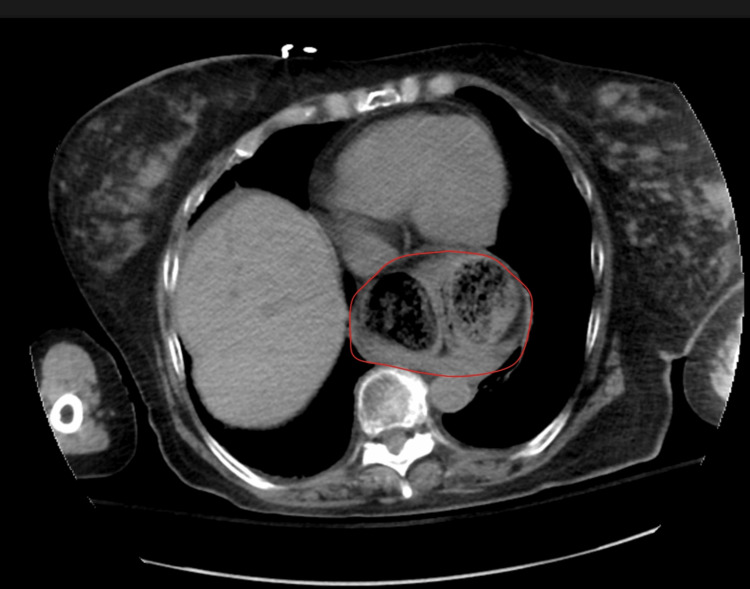
Transverse view of a CT abdomen and pelvis above the diaphragm level showing the apex of the sigmoid volvulus within the thoracic cavity, compressing the heart. The large bowel within the thorax is delineated in red. CT: computed tomography

A computed tomography (CT) scan of the abdomen and pelvis without contrast was suspicious for a sigmoid volvulus with an extension of the apex of the volvulus going through the aortic hiatus and forming a hiatal hernia sac (Figure 2). The proximal volvulus showed extrinsic limited compression at the level of the aortic hiatus (Figures 3-5). Because this patient fit the four Systemic Inflammatory Response Syndrome (SIRS) criteria, she was deemed unstable. She was taken for an emergent laparotomy for a suspected incarcerated Type 4 hiatal hernia complicated by sepsis [5].

**Figure 6:**
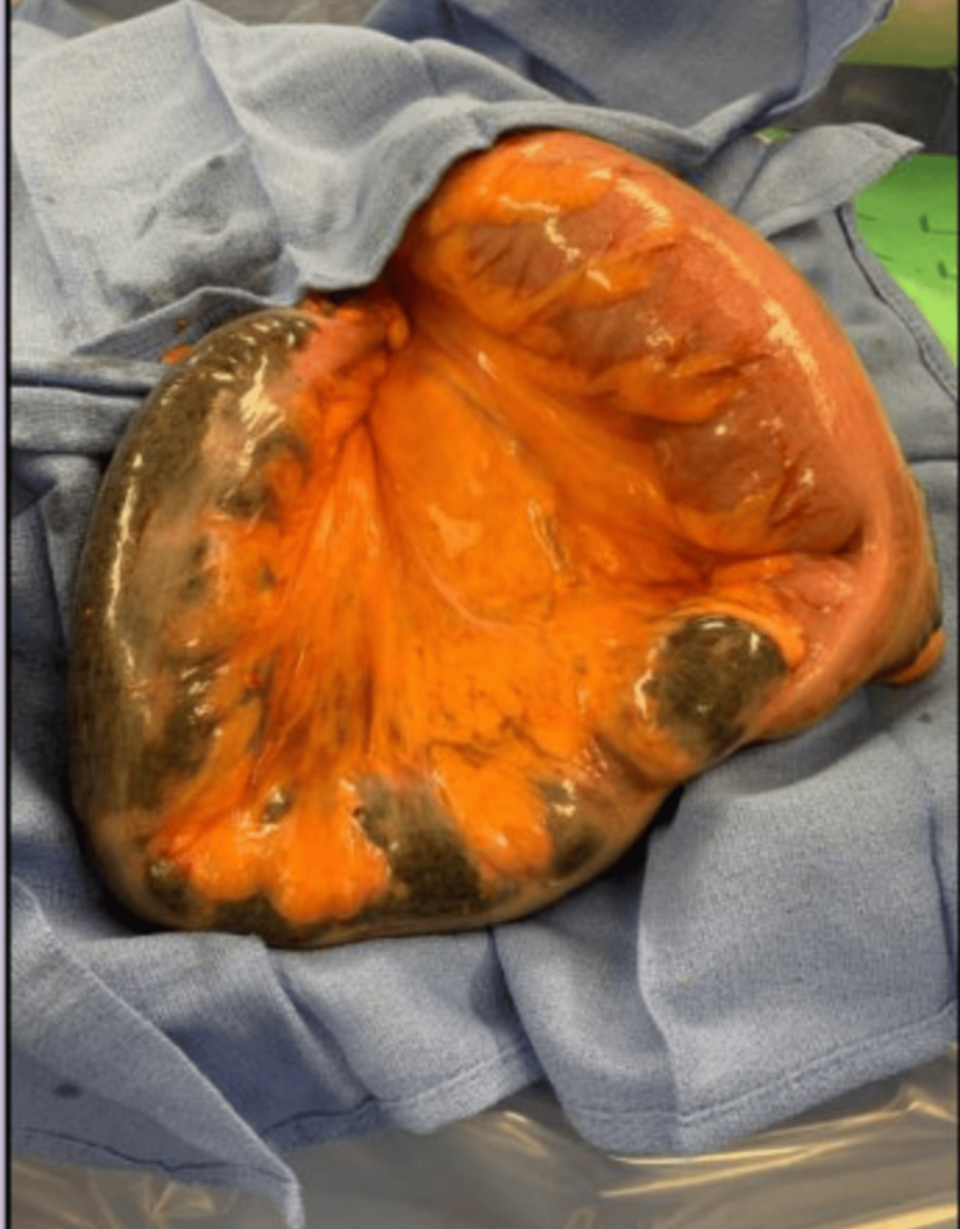
Intraoperative image of a necrotic sigmoid colon reduced from hiatal hernia.

We performed an emergent laparotomy under general anesthesia. Immediately upon entering the peritoneum, we observed the presence of yellow, murky fluid. Cultures were drawn, and the remaining fluid was suctioned using a pool suction. The total amount of fluid was approximately 2-2.5 L. There was also a dilated and necrotic large bowel present that appeared to be a redundant sigmoid. The sigmoid was reduced out of the hiatal hernia, which showed further areas of necrosis (Figure 6). The decision was then made to resect the redundant sigmoid, including all areas of necrosis, create an end colostomy, and leave a distal stump. We decided not to fix the hiatal hernia because the patient was unstable on two pressors and hypothermic. We briefly attempted to explore the hiatus to determine whether we could establish the size of the hernia; however, we could not fully explore it due to instability. The patient was kept intubated postoperatively and transferred to the ICU. She was extubated on postoperative day five and was stable enough for discharge on postoperative day 15. The patient did well with follow-up and was determined to be a good candidate for colostomy reversal based on a colonoscopy two months after discharge. She tolerated the colostomy reversal well and was discharged on postoperative day four with no complications.

## Discussion

Hiatal hernias are reported to affect approximately 15%-20% of the Western population [3]. They can be classified into four different categories. The most commonly diagnosed is Type 1, also known as the “sliding” hiatal hernia, appropriately named because the GE junction is displaced superiorly. Type 2 has the GE junction in its normal anatomical position, but the stomach herniates into the thorax next to the esophagus. Type 3 is a combination of Types 1 and 2, involving displacement of the GE junction and stomach herniation into the thoracic cavity. Our case presentation involves a Type 4 hiatal hernia, the rarest form, which is defined as herniation of both the GE junction and abdominal viscera, not including the stomach, into the thoracic cavity. Specifically, we report intrathoracic herniation of the large distal bowel and sigmoid volvulus, complicated by sepsis due to intestinal incarceration and necrosis.

We conducted a comprehensive literature review from 2015 to the present using the NIH database and found 26 cases of sporadic Type 4 hiatal hernias, which are consolidated into Table 1. Three cases are included for reference in Table 1 but were not included in our analysis because they were pediatric cases likely due to congenital defects. The patients presented with a diverse number of symptoms in addition to classic gastrointestinal dysfunction, such as chest pain, dyspnea, retrosternal pressure, and dysphagia. Of the cases included in our review, nine patients exhibited the listed nonclassic symptoms. Chest pain was a common symptom due to the mass effect and was present in 22% of cases. Similarly, shortness of breath was frequently reported, as demonstrated in 39% of the included transcripts. These data emphasize the need for careful diagnosis and a thorough workup because the progression of symptoms can be life-threatening. Pneumothorax, intestinal perforation, and shock are only some examples of fatal complications listed in Table 1.

**Table 1:** A summary of Type IV hiatal hernia from a literature review starting from 2015 to 2024. *Pediatric cases included for reference but not considered in our analysis, most likely due to congenital diaphragmatic defects

Year	Author	Age	Gender	Symptom	Treatment	Involved Organ	Hernia Repaired	Complication
2016	Patel [6]	65	M	Epigastric pain, vomiting	Conservative	Stomach, duodenum, body and tail of the pancreas	No	Acute pancreatitis
2016	Fam [7]	46	M	Chest pain, dyspnea	Anti-ischemic therapy, conservative	Stomach, body and tail of the pancreas	No	None
2017	Rendon-Medina [8]	59	F	Abdominal pain, vomiting, obstipation, dyspnea	Laparoscopy, Nissen type fundoplication without Collis gastroplasty	Stomach, greater omentum, transverse colon	Yes	None
2017	Wang [9]	102	F	Chest pain, nausea, vomiting, dyspnea	Supportive	Stomach, duodenum, body and tail of the pancreas	No	Pancreatitis
2017	Biswas [10]	90	M	Abdominal pain, nausea, vomiting	Laparotomy	Stomach, jejunum	Yes	Jejunal diverticula perforation
2018	Erdas [11]	71	M	Abdominal pain, dyspnea, nausea	Laparoscopic repair of left diaphragmatic hernia	Small bowel, transverse colon, greater omentum	Yes	Left pneumothorax
2018	Mittal [12]	71	M	Acid reflux, vomiting, retrosternal burning	Laparoscopic repair	Stomach, transverse colon, greater omentum	Yes	None
2019	Zackria [13]	33	M	Epigastric pain, chest pain, dyspnea, nausea, vomiting	Laparotomy	Stomach, body of pancreas	Yes	Acute pancreatitis
2019	Yuda Handaya [14]	70	F	Abdominal pain, dyspnea	Laparotomy, hernia repair with self-attached mesh	Stomach, ileum, transverse colon, omentum	Yes	None
2019	Umemura [15]	74	F	Dyspnea, severe anorexia, chest pain	Laparoscopic Nissen procedure	Stomach, jejunum, pancreas	Yes	Intrathoracic organoaxial gastric volvulus
2020	Zain [16]	8	M	Epigastric pain, vomiting	Laparotomy	Stomach, small bowel, transverse colon	Yes	Gastric volvulus
2020	Tomida [17]	86	F	Epigastric pain, vomiting	Laparotomy with Toupet fundoplication	Stomach, transverse colon, pancreatic body and tail	Yes	Damage to pleura during surgical intervention requiring tracheal tube
2020	Tustumi [18]	38	F	Nausea, vomiting	Pre-op botulinum toxin, laparoscopic hiatoplasty with anterior partial fundoplication and gastropexy	Stomach, small bowel, unspecified colon, spleen	Yes	None
2020	Konno-Kumagai [19]	65	F	Anorexia	Laparotomy, partial transverse colon resection, colostomy	Stomach, transverse colon	Yes	Septic shock, acute mediastinitis
2021	Rassam [20]	63	M	Retrosternal pressure	Laparoscopic omentum repositioning with hiatoplasty	Greater omentum	Yes	Complete esophageal obstruction; repositioning of greater omentum
2021	Plourde [21]	Unknown	F	Abdominal pain, nausea	Laparotomy with partial small bowel and colon resections	Small bowel, transverse and left colon	Yes	Obstructive shock, sepsis
2021	Hou [22]	77	F	Malaise, anorexia, diarrhea, dyspnea	Conservative	Stomach, large intestine	No	None*
2022*	Tariverdi [23]	9	M	Abdominal pain, vomiting	Laparotomy	Stomach, colon, spleen	Yes	None
2022*	McDowell [24]	7d	F	Abdominal distension, vomiting	Laparoscopy	Gastric antrum, pylorus, duodenum	Yes	None
2022	Gielis [25]	29	M	Abdominal pain	Laparoscopy	Stomach, greater omentum, transverse colon	Yes	None
2022	Yoneda [26]	85	M	Dysphagia	Hiatal hernia repair followed by distal pancreatectomy	Stomach, pancreas	Yes	Gastric stasis
2022	Mejri [27]	56	F	Abdominal pain, vomiting, chest discomfort	Conservative	Stomach, transverse and right colon, duodenum, pancreas	No	Acute pancreatitis with herniation
2023	Lin [28]	77	F	Postprandial abdominal pain, nausea, vomiting	Laparoscopic hiatal hernia repair and Nissen fundoplication	Antrum, proximal duodenum	Yes	Mesenteroaxial gastric volvulus
2023	Zhu [29]	60	M	Epigastric pain, abdominal distension, vomiting	Laparotomy, splenectomy, gastropexy	Stomach, colon, spleen, pancreas, greater omentum	Yes	Gastric volvulus
2023	Ludena [30]	55	F	Epigastric pain, dyspnea, nausea, vomiting	Laparotomy, hiatoplasty	Stomach, transverse colon, greater omentum, left hepatic segment	Yes	Transverse colon volvulus, hydropneumothorax
2023	Inoue [31]	77	F	Vomiting	Laparoscopic Toupet fundoplication	Esophagus, stomach, duodenum	Yes	Incarceration of the duodenal bulb

The classic presentation of a hiatal hernia would be severe abdominal or epigastric pain, heartburn, nausea, vomiting, postprandial pain, and anorexia. If such symptoms are paired with a symptom caused by a mass effect from increased intrathoracic pressure, such as trouble breathing or chest pain, a severe hiatal hernia should be considered. Many diagnostic tools are available to aid in confirmation. Plain chest radiography will typically show a retrocardiac gas or fluid level, representing an intrathoracic stomach or intestine. Obtaining a CT scan is helpful in determining herniation content, size, and location and should be considered the gold standard for diagnosis [32]. Monitoring vital signs, complete blood cell counts, and comprehensive metabolic panels should also be considered to help provide a better clinical picture. In our patient, they helped diagnose sepsis, which was critical information that guided our treatment plan. Some have reported the use of inflammatory markers such as C-reactive protein tests and procalcitonin levels as helpful aids in diagnosis. Increased levels can indicate an active inflammatory process [33,34].

Many life-threatening complications can arise from a Type 4 hernia, such as strangulation, gastric volvulus, and sepsis, but less severe complications have also been noted, such as pancreatitis or dysphagia, all of which are listed in a separate column in Table 1. The case our patient presented is a rare one, not only because of the incarceration of the bowel, which caused preoperative sepsis, but also because herniation of the sigmoid colon into the thorax is a highly uncommon occurrence. In the cases included in our review, 52% of herniations involved the small bowel, 35% of patients exhibited pancreatitis due to a herniated pancreas, 9% included splenic herniation, and 61% involved the large intestine. Of the cases that involved the large intestine, 79% included the transverse colon, and only one other case went as far as the descending colon. None included the sigmoid colon or showed evidence of sigmoid volvulus. The cause of the herniation was unclear, but based on one case report, it is possible that compromise of the ligaments stabilizing the intestines could have contributed to the severe displacement of the most distal part of the colon [22].

Our patient did not undergo repair of the hiatal hernia, but there has been much debate on indications and the risks of hiatal hernia repair, specifically for Type 4 hernias. Of all the reports included in our review, only 22% shared the same course and deferred repair. The choice between emergent, elective repair, and staged or semi-elective surgery should be carefully considered. Not surprisingly, the risk of postoperative complications, prolonged hospital stays, and severe complications increases significantly among emergent cases. It should be noted that these cases are typically far more severe. When researchers controlled for significant comorbidities that affect the risk of surgical intervention in general, the difference was no longer significant [35]. This suggests that complications are more dependent on patient comorbidities than on urgency. Another study took this finding further and found that frailer patients and preoperative sepsis increased the odds of mortality, whereas a laparoscopic approach, as compared to open exploratory laparotomy, and a BMI ≥ 25 as compared to a BMI < 18.5 were protective of mortality [36]. When we specifically consider these factors in our patient, she fits all categories for a high risk of complications and a complicated hospital stay, which further supports our decision to defer repair.

In non-emergent cases, the gold standard, according to the Society of American Gastrointestinal and Endoscopic Surgeons, is to repair all symptomatic paraesophageal hiatal hernias, and an essential measure during repair is to return the GE junction to an infradiaphragmatic position and reinforce it with mesh for larger hernias [37]. Although these guidelines should be considered for incidental hiatal hernia diagnosis, our report features an uncommon situation. A case-by-case analysis should be considered for each patient when making recommendations for repair based on the series of diagnostic tools previously highlighted.

## Conclusions

Type 4 hiatal hernias are rare entities but are typically the most severe. Patient presentations vary because of the herniation into the thoracic cavity, leading to a multitude of clinical symptoms and, often, a delay in diagnosis. Thus, these cases frequently involve life-threatening complications and require emergent surgical intervention. Comprehensive evaluation is imperative for early intervention and the prevention of progression to hemodynamic instability.
